# Unlocking protein-based biomarker potential for graft-versus-host disease following allogenic hematopoietic stem cell transplants

**DOI:** 10.3389/fimmu.2024.1327035

**Published:** 2024-02-16

**Authors:** Maria Iacobescu, Cristina Pop, Alina Uifălean, Cristina Mogoşan, Diana Cenariu, Mihnea Zdrenghea, Alina Tănase, Jon Thor Bergthorsson, Victor Greiff, Mihai Cenariu, Cristina Adela Iuga, Ciprian Tomuleasa, Dan Tătaru

**Affiliations:** ^1^ Department of Proteomics and Metabolomics, MEDFUTURE Research Center for Advanced Medicine, “Iuliu Hatieganu” University of Medicine and Pharmacy, Cluj-Napoca, Romania; ^2^ Department of Pharmacology, Physiology and Pathophysiology, Faculty of Pharmacy, “Iuliu Hatieganu” University of Medicine and Pharmacy, Cluj-Napoca, Romania; ^3^ Department of Pharmaceutical Analysis, Faculty of Pharmacy, “Iuliu Hatieganu” University of Medicine and Pharmacy, Cluj-Napoca, Romania; ^4^ Department of Translational Medicine, MEDFUTURE Research Center for Advanced Medicine, “Iuliu Hatieganu” University of Medicine and Pharmacy, Cluj-Napoca, Romania; ^5^ Department of Hematology, Faculty of Medicine, “Iuliu Hatieganu” University of Medicine and Pharmacy, Cluj-Napoca, Romania; ^6^ Department of Stem Cell Transplantation, Fundeni Clinical Institute, Bucharest, Romania; ^7^ Department of Laboratory Hematology, Stem Cell Research Unit, Biomedical Center, School of Health Sciences, University Iceland, Reykjavik, Iceland; ^8^ Department of Immunology, University of Oslo, Oslo, Norway; ^9^ Department of Animal Reproduction, University of Agricultural Sciences and Veterinary Medicine, Cluj-Napoca, Romania; ^10^ Department of Internal Medicine, Faculty of Medicine, “Iuliu Hatieganu” University of Medicine and Pharmacy, Cluj-Napoca, Romania

**Keywords:** allogenic stem cell transplantation, graft-versus-host-disease, biomarker, proteomics, biofluids

## Abstract

Despite the numerous advantages of allogeneic hematopoietic stem cell transplants (allo-HSCT), there exists a notable association with risks, particularly during the preconditioning period and predominantly post-intervention, exemplified by the occurrence of graft-versus-host disease (GVHD). Risk stratification prior to symptom manifestation, along with precise diagnosis and prognosis, relies heavily on clinical features. A critical imperative is the development of tools capable of early identification and effective management of patients undergoing allo-HSCT. A promising avenue in this pursuit is the utilization of proteomics-based biomarkers obtained from non-invasive biospecimens. This review comprehensively outlines the application of proteomics and proteomics-based biomarkers in GVHD patients. It delves into both single protein markers and protein panels, offering insights into their relevance in acute and chronic GVHD. Furthermore, the review provides a detailed examination of the site-specific involvement of GVHD. In summary, this article explores the potential of proteomics as a tool for timely and accurate intervention in the context of GVHD following allo-HSCT.

## 1 Background on graft-versus-host-disease

In hematology, precision medicine has made strides, particularly in bone marrow transplants (BMT) and targeted therapies transforming blood disorder management. BMTs recent safety profile, with transplant-related mortality below 30% and a 30 to 70% risk of graft-versus-host disease (GVHD), positions it as a universal option. However, advances in genetics, molecular biology, and immunology have led to targeted therapeutics, reducing BMT necessity in some cases.

Currently, allogenic hematopoietic stem cell transplants (allo-HSCT), using bone marrow, peripheral blood stem cells (PBSCs), or cord blood, are increasingly performed globally. Allo-HSCT holds a great potential of curing many malignant and non-malignant hematologic disorders, subjected to specific conditions (1). Risk factors include human leukocyte antigen (HLA) mismatch, female donors for male recipients, and total body irradiation. Complications post-allo-HSCT involve infections, GVHD, and relapse, with risk assessment relying on clinical characteristics (2, 3).

GVHD is a life-threatening complication that can occur after the allo-HSCT. About 30 to 80% of allo-HSCT recipients develop acute GVHD (aGVHD) and around 50% chronic GVHD (cGVHD) (4). In this context, GVHD arises when immunocompetent T cells from the donated tissue (the graft) identify the recipient (the host) as foreign. This recognition triggers an immune response that activates donor T cells, enabling them to develop cytolytic capacity and attack the recipients cells carrying foreign antigens (5). In hematological malignancies, there is a fine balance between the harmful consequences of GVHD and the therapeutic effects produced when donor lymphocytes attack recipient malignant cells, in graft versus leukemia/tumor (GVL) effect.

In aGVHD, the skin, liver or gastrointestinal tract can be affected in about 2 to 12 weeks after allo-HSCT, but there are situations when aGVHD appears before or after this interval. A cascade of events initiated by the activation of innate immune cells and subsequent tissue injury induces aGVHD, which is additionally exacerbated by the engagement of adaptive immune responses. The hallmark of aGVHD is the infiltration of activated donor T cells, predominantly CD4+ cells, into host tissues, driven by their recognition of the recipients tissues as allo-antigenic. This process results in severe inflammation that further amplifies the immune response, leading to a cytokine storm. In cytokine storm, elevated levels of circulating cytokines and immune cell hyperactivation occurs. The intricate interplay between innate and adaptive immunity during aGVHD underscores the multifaceted nature of this condition, necessitating a comprehensive understanding of the underlying immunological mechanisms for the development of effective therapeutic strategies. The dynamics of aGVHD pathogenesis, particularly the contributions of specific immune cell subsets and the cytokine storm, is crucial for both understanding the disease and for identifying new biomarkers to help early diagnosis, prognosis and targeted interventions to improve clinical outcomes.

Additionally, cGVHD can develop within 4 to 6 months post-allo-HSCT, following aGVHD or in patients without any prior complications. This condition is similar to an autoimmune disease, involving, besides donor T cells, extensive B cell activation, the release of pro-inflammatory cytokines that can affect the skin, mouth, eyes, gastrointestinal tract and the liver. The pathophysiology of cGVHD involved 3 stages (1): high production of inflammatory cytokines (interleukin-1 (IL-1), IL-6, TNF-α) and expression of antigens of the major histocompatibility complex (MHC) that stimulate antigen-presenting cells to interact with the donor T cells (2), this interaction leads to the activation of T cells which then release additional inflammatory cytokines (IL-2, Interferon- γ (INF-γ)) and (3) the migration of cytotoxic T lymphocytes and natural killer cells to specific organs, producing tissue damage (6).

GVHD is an important cause of non-relapse mortality (NRM), accounting for about 11% deaths in patients receiving allo-HSCT (7). The need for risk stratification for GVHD is high, due to problems associated with immunosuppression. It is either not aggressive enough leaving patients with a high GVHD risk vulnerable, or it is too aggressive leading to infections and other complications (3, 8).

A major concern consists in the development of tools capable of (i) timely identification of patients at risk for GVHD, (ii) invasive procedures avoidance and (iii) timely intervention, one such option could be a biomarker. Currently, there is no validated specific biomarker for GVHD. In the clinical scenario the diagnosis is confirmed through analysis of the biospecimen of the involved site.

As such, fast and organ-specific diagnosis of GVHD based on biomarkers can lead to targeted interventions, and prognosis biomarkers can assist in the process of decision making for treatment. Until recently, biomarkers were identified by hypothesis-driven approaches, considering the pathophysiologic role of specific molecules in GVHD, such as miRNAs and cellular biomarkers for GVHD (2, 9).

The aim of this paper is to review the potential candidate biomarkers derived from unbiased approaches, facilitated by the recent advancements in proteomics a robust high-throughput technique. These biomarkers may or may not have a direct pathophysiologic link to GVHD; instead, they might emerge due to the condition itself or other unexplored interactions.

## 2 Proteomics as the key strategy for maximizing minimally invasive biopsies towards biomarker discovery

A biomarker is defined by the Food and Drug Administration - National Institute of Health (FDA-NIH) Biomarker Working Group as “a characteristic that is measured as an indicator of normal biological processes, pathogenic ones, or a response to an exposure or intervention” (10). Biomarkers have diverse applications, guiding healthcare interventions for maximum patient advantage. They are commonly utilized for disease diagnosis, risk-based population screening, evaluating disease complications and therapy effectiveness. Furthermore, they facilitate disease trajectory prediction, aid therapy selection, and assess novel drug effectiveness and safety. Thus, biomarkers can be diagnostic biomarkers, prognostic biomarkers and theranostic biomarkers (11).

An ideal biomarker should be easily quantifiable, allowing for simple procurement of biological specimens and employing a financially viable quantification method. It is crucial for a biomarker assay to demonstrate reproducibility, sensitivity (SEN), and specificity (SPE), providing accurate results for clinicians to make informed therapeutic decisions. The efficacy of a biomarker depends on its strong correlation with treatment responses. To establish clinical applicability, a comprehensive series of assays in both preclinical and clinical settings is necessary. Following the identification of a prospective biomarker candidate, validation processes, rigorous evaluation, and refinement through clinical investigations are conducted before approval and commercialization (11).

In the development of GVHD biomarkers, optimizing statistical test performance and determining cut points are critical. *Bidgoli et al.* (12) emphasized the importance of Receiver Operating Characteristic (ROC) curves and Area Under the Curve (AUC) in assessing assay performance. SEN and SPE, represented by ROC curves, reflect true positive and true negative rates, respectively. Positive and negative predictive values are calculated considering both cut points and GVHD incidence, with the latter being particularly relevant in HCT. Tailoring biomarker cut points for clinical use involves testing multiple values to balance intervention efficacy and toxicity. Hazard ratio (HR) is another key parameter used to gauge the impact of an intervention on a specific outcome over time. In biomarker discovery, HR helps evaluate the link between a biomarkers expression and the timing of an event, suggesting its potential as a prognostic indicator (13). Clinical trials using biomarkers for patient selection have a higher likelihood of success compared to those lacking biomarkers (14).

### 2.1 Proteins as a valuable biomarker source

Despite advances in genomics and technology, clinical biomarker discovery faces persistent challenges. Solely focusing on genetic abnormalities does not fully capture the complexity of disease biology, involving interactions between genetic anomalies, epigenetics, post-translational modifications, and immune responses (15). Proteins, as structural and signaling entities, are pivotal in understanding disease characteristics and serve as key drug intervention targets. Exploring the proteome, representing the entire set of proteins expressed under specific conditions, provides valuable insights into their functions, interactions, and roles in biological systems. Notably, identifying proteins and monitoring their levels in biological samples dynamically reflects the cumulative impact of genetic and epigenetic changes, crucial for understanding functional disease biology and guiding therapeutic strategies (16, 17).

Exploring biomarkers from both membrane-bound and soluble protein forms is intriguing. Membrane-bound proteins are crucial for signaling, transport, and adhesion, with changes indicating disease signatures (18). Simultaneously, measuring soluble proteins in bodily fluids offers a non-invasive and dynamic approach for biomarker discovery, providing real-time insights into physiological states. This versatile method applies across diseases, identifying diagnostic, prognostic, and therapeutic indicators. Soluble biomarkers, exemplified by soluble immune checkpoints, have emerged as dynamic molecules with significant physiological roles. The intricate network of immune checkpoints, encompassing diverse stimulatory and inhibitory pathways, plays a pivotal role in fine-tuning immune responses. Notably, molecules like Programmed Cell Death Protein 1 (PD-1), Cytotoxic T-lymphocyte associated protein 4 (CTLA4), and their ligands have emerged as focal points for anti-cancer immunotherapy, while other checkpoint pathways contribute to the immunopathogenesis of human diseases as critically analyzed by *Riva A *(19). Beyond the traditional view of membrane-bound systems, immune checkpoints also manifest as soluble forms (sCRs), actively regulating immune responses both locally and systemically. In a comprehensive review by *Niu et al.* (20), the roles of sPD-1 and sPD-L1 in cancer are explored, shedding light on their synthesis, release, and alternative mRNA splicing mechanisms. The study suggests that PD-1 may function as an immune stimulator, in contrast to its membrane-bound counterpart, while sPD-L1 retains suppressive activity. The review also proposes the intriguing idea that sCRs could act as decoy sponges for therapeutic blocking antibodies, impacting resistance to immunotherapy and disease progression. The complexity of sCR networks, disease-specific signatures, and the persistence of sCR “imprinting” post-treatment pose challenges (21–24). The articles collectively support the hypothesis that soluble isoforms of immune checkpoints may exhibit distinct functions compared to their membrane-bound counterparts. Furthermore, the existence of various isoforms for each sCR and soluble receptor-ligand complexes underscores the need for standardized measurement criteria and open comparisons of antibody clones. In conclusion, unraveling the intricacies of the sCR system holds promise for advancing both basic and translational research, providing valuable insights into their roles as disease biomarkers and potential targets for pharmacological interventions and immunotherapy. Nevertheless, analyzing both forms enhances our understanding of disease biology, supporting precision medicine. This dual approach boosts the potential for discovering robust biomarkers, influencing disease diagnosis, prognosis, and treatment (12).

### 2.2 Proteomics as a discovery instrument

Proteomics, an ‘omics strategy, is crucial for profiling and quantifying proteins in a biospecimen under specific conditions. This involves investigating protein abundances, protein-protein interactions, and the functional roles of proteins in diverse conditions. Proteomics methodologies are classified as low-throughput and high-throughput. Low-throughput approaches include antibody-based techniques like Enzyme-Linked Immunosorbent Assay (ELISA) and Western blotting (WB), relying on antigen-antibody interactions for protein detection. Electrophoresis methods, such as two-dimensional gel electrophoresis and sodium dodecyl-sulfate polyacrylamide gel electrophoresis (SDS-PAGE), separate proteins based on characteristics like mass, charge, or isoelectric point, followed by identification using molecular weight standards or mass spectrometry (MS). Chromatography, another separation technique, aids in isolating and purifying proteins through ion-exchange, size exclusion, and affinity chromatography. Downstream analyses, including MS, contribute to protein identification and quantification post-separation (25).

**Figure 1:**
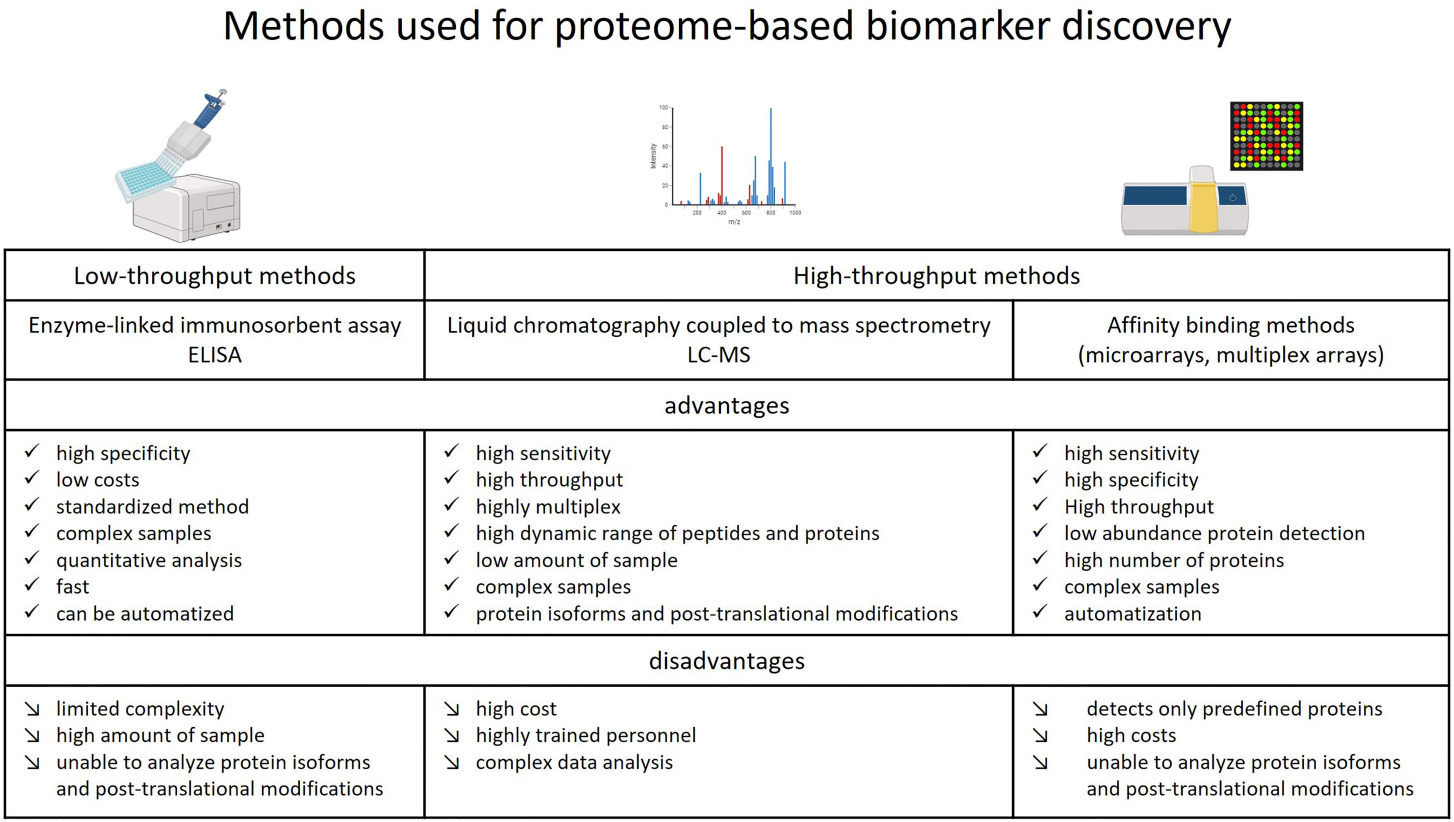
Advantages and disadvantages of methods used for proteome-based biomarker discovery studies for GVHD.

High-throughput methodologies in proteomics involve affinity binder techniques and MS. Affinity binder techniques, such as microarrays and multiplex arrays, use specific antibodies and aptamers to capture target proteins (26). Microarray research, particularly in the context of cancer and hematologic disorders, has identified candidate diagnostic biomarkers for GVHD. MS, employed in both top-down (intact protein analysis) and bottom-up (protein digestion to peptides before analysis) settings, involves ionization, separation, and detection of proteins or peptides. Ionization modes like electrospray ionization and matrix assisted laser desorption/ionization (MALDI), along with mass analyzers like time-of-flight and quadrupole, contribute to accurate protein identification, including isoforms and posttranslational modifications. MS-based protein quantification, especially with tandem MS (MS/MS), is highly accurate and relies on mass spectra matched to sequence databases (27). Best practices for protein-based biomarker discovery and validation are discussed elsewhere (28). The preferred high-throughput approaches for biomarker discovery, including those for GVHD, involve bottom-up proteomics and affinity-based methods (29). **Figure 1** illustrates the advantages and disadvantages of these proteomics methods in the context of GVHD biomarker discovery. Proteomics, as a prominent ‘omics technology, has witnessed significant progress, overcoming challenges and contributing to the pursuit of protein-based biomarkers for precision medicine in recent years (30).

### 2.3 Biomarker panels

Efficiently measuring multiple markers can be achieved through multiplexing techniques, which allow simultaneous detection of various biomolecules in a single assay. Multiplexing enhances throughput, conserves sample volume, and reduces costs compared to running individual assays for each marker. Technologies such as multiplex immunoassays, microarrays, and MS enable the detection of multiple markers within a single experimental run. Additionally, advances in high-throughput screening methods and automation contribute to efficiency by streamlining the workflow. Choosing an appropriate multiplexing platform based on the characteristics of the markers and the research objectives ensures a comprehensive and resource-effective approach to measuring multiple biomarkers simultaneously (31).

Measurements of individual candidate levels are not always relevant or specific. That is why their use in combination of multi-marker panels can bring an increase in SEN and SPE. MS based proteome studies towards biomarker discovery for GVHD accurately distinguished GVHD samples from both posttransplant non-GVHD samples and pretransplant samples. Furthermore, distinct serum proteomic signatures were identified to distinguish pretransplant from posttransplant non-GVHD samples, opening up new insights towards biomarker panel discoveries (32). For aGVHD protein-based biomarker panel discovery, saliva and plasma were the non-invasive biofluids investigated and the identified panels are further presented.

### 2.4 Valuable biomarker sources

Proteome analysis of biospecimens offers insights into physiological processes, aiding the identification of actionable approaches, including biomarkers applicable at the bedside. Primary sources of protein markers include tissues and body fluids such as serum, plasma, saliva, urine, and bone marrow. While bone marrow provides heightened diagnostic precision, non-invasive biospecimens, like plasma and serum, present advantages in terms of patient compliance and reduced procedural risks.

Plasma and serum, being popular biofluids for biomarker discovery, offer easy acquisition and broad molecular representation due to systemic circulation. Challenges like sample standardization and accessing low-abundance proteins can impact proteomics studies, but the Human Proteome Organization (HUPO) provides Biomarker Discovery Protocols for addressing these issues (33). Tears, a non-invasive biospecimen, are gaining attention, particularly for ocular involvement in GVHD. In gastrointestinal GVHD, feces can serve as a useful biospecimen, providing insights into intestinal inflammation. Skin biopsies, obtained minimally invasively, are valuable for identifying specific biomarkers related to skin manifestations of GVHD. Urine, an alternative to blood samples, offers advantages such as ease of collection, larger quantities, and a less complex protein mixture with low abundance variation. However, its limitation lies in providing information primarily about diseases in the organs directly involved in its production and excretion, like the kidneys, rather than systemic diseases.

Proteomics, aiming to translate discoveries into clinical practice, focuses on uncovering minimally invasive biofluid-based biomarkers (34, 35). Examples of biospecimens for GVHD biomarker studies are detailed in the following sections.

## 3 Protein-based biomarkers for GVHD following allo-HSCT

Technological advancements have facilitated the comprehensive profiling of proteins within a given biological system. Unbiased investigations have yielded intriguing findings that might have been overlooked using narrower analytical approaches. Diverse biomarkers, when evaluated prior to BMT, play a pivotal role in the selection of suitable candidates for intervention, particularly in cases of unfavorable prognostics, resistance to chemotherapy, or heightened susceptibility to drug-induced toxicities. Furthermore, post- HSCT biomarkers offer valuable information regarding prognosis and the likelihood of complications. The diagnosis of GVHD is still dependent on clinical criteria in most of the part and requires the realization of skin biopsy, colonoscopy, bronchoscopy and bronchoalveolar lavage. aGVHD is the main cause of morbidity after transplant, being responsible for 15 to 40% of the post-transplant deaths. Mortality in GVHD is caused by the difficulty in giving a diagnosis and planning the optimal moment for therapy start (1, 36). As previously mentioned, GVHD markers can facilitate quick diagnosis of this complication and quick start of the therapy, which would increase the survival rate. Also, based on stratification biomarkers, the patients can get a more or less aggressive immunosuppressive regime reducing the costs and the long-term toxicity (34). Different strategies have been approached towards GVHD biomarker discovery targeting proteins. Strategies include single biomarker as well as multi-marker panel scouting and using different biospecimen. A comprehensive overview is further presented.

### 3.1 Protein based biomarkers for aGVHD

#### 3.1.1 Cytokine-based biomarkers for aGVHD

Cytokines are important in both protection after HSCT, notably in graft-versus-leukemia and in disease, notably in GVHD. Acute GVHD is a complex pathologic process that involves many types of T-cells that secrete cytokines: Th1, Th2, TH17 and CD8+ T cells. Most cytokines associated with aGVHD include ILs, TNF-α and IFN-γ. Many cytokines are also important in the inflammatory cytokine storm that is specific to aGVHD, including the proinflammatory cytokines IL-12, IL-17, IL-21, IL-33, and the protective cytokines IL-10, IL-22, and TGF-β (37). Even more, the involvement of soluble interleukin-2 receptor (sIL-2R) in the physiopathology of GVHD has led to the development of targeted therapies with monoclonal antibodies, like daclizumab and conjugates like denileukin diftitox (38). The administration of anti-IL-6 receptor antibodies was associated to a drop in GVHD mortality (39). Thus, because of the role of cytokines in systemic inflammation during aGVHD, they were studied as potential biomarkers with diagnostic or predictive value. Mostly, they were studied from serum or plasma by employing ELISA methods. The cytokine-based biomarkers for aGVHD are presented in **Table 1**.

**Table 1:** Cytokine-based biomarkers for aGVHD.

Cytokine	Specimen	Method	Clinical relevance	SEN/SPE	Ref.
Diagnostic biomarker					
IL-7	serum	ELISA	Raised serum IL-7 at 7 to 14 days after transplant are associated with aGVHD.	86/100	(40)
sIL-7, sIL-7R	plasma	ELISA	Reduced sIL-7R levels may indicate an elevated risk of GVHD, suggesting insufficient availability for the IL-7 ‘buffer system. Assessing plasma sIL-7R levels, along with IL-7, could help identify individuals at higher risk for GVHD and potentially CMV infection.	–	(41)
IL-8, IFN-γ	plasma	ELISA	Reduced levels of IL-8 on day +7 and IFN-γ post-engraftment were linked to the occurrence of grade II-IV aGVHD and severe aGVHD. Additionally, decreased IFN-γ after engraftment was connected to a higher risk of NRM.	–	(42)
IL-12, IFN-γ	mononuclear cells	microbead array technology	Donor IL-12 and IFN-γ were associated with aGVHD.	IL-12: 100/50 INF- γ: 96.8%/-	(43)
Prognostic biomarker					
IL-18	serum	ELISA	Increased pre-transplant levels of free IL-18 correlated with lower risks of both NRM (HR 1.24) and overall mortality (HR 1.22) following allo-HSCT.	–	(44)

SEN, sensitivity; SPE, specificity; -, not available; CMV, Cytomegalovirus infection; HR, hazard ratio; Ref, reference.

#### 3.1.2 Other emerging protein-based biomarkers for aGVHD

In addition to cytokines, extensive research has been conducted on various protein-based biomarkers, and their promising utility as potential indicators of aGVHD is currently on the rise. The suppression of tumorigenicity 2 (ST2) is a receptor included in the IL-1 family, which binds specifically to IL-33. ST2 has two isoforms: a transmembrane (mST2) and a soluble form (sST2). During aGVHD, an increase in IL-33 has been observed, leading to specific inflammation and tissue damage (45). The sST2 receptors are expressed in different cells and act as decoy receptors, sequestering free IL-33, thereby preventing IL-33-mediated proinflammatory actions (46). Studies have found an increase of sST2 which could be correlated with GVHD severity in patients. A possible explanation given by earlier studies was that the release of sST2 in the serum occurs very late in the inflammatory response resulting in the inability of sST2 to sequester circulating IL-33. This could make sST2 an interesting biomarker for aGVHD and NRM (47). Also, *Vander Lugt et al.* found by quantitative liquid chromatography coupled with MS/MS and further ELISA validation that ST2 could be a prognostic marker for GVHD with endpoints such as treatment resistance or death 6 months after therapy, with SEN and SPE for six-month post-transplant NRM in an independent set of 70 and 64 respectively (48).

Another marker is vascular endothelial growth factor (VEGF). Studies that have evaluated the prognostic value of VEGF levels in GVHD presented with controversial results (49). Some studies have reported that high VEGF levels were associated with the development or severity of GVHD in a mouse model study (50), whereas others have found that VEGF either has a protective effect against severe aGVHD (AUC 0.69) (51) or no correlation with GVHD (52). Low VEGF levels after allo-HSCT were found to be associated with NRM due to severe exacerbation of aGVHD, implying the biomarker utility of VEGF. VEGF monitoring after allo-HSCT might identify those patients at risk of severe transplant-related mortality (53).

Another interesting molecule is T cell immunoglobulin and mucin domain 3 (TIM3). TIM3 is a transmembrane receptor protein expressed on T cells that produce IFN-gamma, Tregs, myeloid cells, natural killer cells, and mast cells, where it inhibits cytokine expression (54). Although the physiopathology concerning the involvement of TIM3 in aGVHD is not fully understood, and only preclinical studies have given hints regarding the regulation of hepatic CD8+ T cells by TIM3 (55), clinical proteomics studies have shown that the levels of the soluble extracellular domain of Tim-3 exhibited an elevation in the bloodstream of individuals preceding the clinical manifestation of aGVHD. Furthermore, these levels demonstrated a correlation with the severity of gut GVHD (AUC of 0.79) (56).

Finally, macrophage migration inhibitory factor (MIF), a pleiotropic protein, is known to participate in inflammatory and immune responses by regulating TNF-α production. Elevated peak serum MIF at acute GVHD onset and increased mean serum MIF in patients developing extensive cGVHD within 6 months suggest heightened MIF levels during active phases of both GVHD types (57).

#### 3.1.3 Salivary protein panels for aGVHD

Towards salivary protein panel discovery, the salivary proteome was analyzed by gel electrophoresis with subsequent MS/MS analysis and ELISA. MS identified significant salivary protein fluctuations lasting at least 2 months posttransplant. High salivary levels of lactoferrin, cystatin SN, albumin and salivary amylase have been identified in patients with GVHD with oral cavity involvement. High levels of these proteins indicate pathophysiological processes that take place in GVHD with oral cavity involvement, like salivary glands infiltration, activation of immunomodulatory processes and increasing the permeability of the oral cavity mucosa and plasma extravasation (58). Also by MS, proteins from the S100 protein family was found to play a role in aGVHD diagnosis: S100A8, S100A7 and S100A9 showing SEN of 60% and SPE of 88% (59).

#### 3.1.4 Plasma protein panels for aGVHD

Another strategy to identify biomarker panels was to screen a large set of proteins by antibody array and to validate the results by ELISA. This was done for four plasmatic diagnostic markers: IL-2-receptor-alpha (IL-2Rα), tumor-necrosis-factor-receptor-1 (TNFR1), IL-8 (IL-8), and hepatocyte growth factor (HGF). The 4 biomarkers panel confirmed their potential for diagnosis of GVHD in patients at onset of clinical symptoms and provided prognostic information independent of GVHD severity with AUC of 0.86 (60). Elafin, a skin-specific marker and regenerating islet-derived 3-α (Reg3α), a gastrointestinal tract-specific marker was added later to the 4-parameter panel. The panel was studied for its potential to discriminate between therapy responsive and nonresponsive patients and predict survival in patients receiving GVHD therapy. Even more, the 6 biomarker panel demonstrated potential to be used for early identification of patients at high or low risk for treatment non responsiveness or death, and provided opportunities for early intervention and improved survival after HSCT (61). Also in pediatric patients with aGVHD a biomarker panel of TNFR1, IL-2Rα, HGF, CCL8, IL-8 and IL-12p70 demonstrated its utility following HSCT (62). Another combination of proteins, namely a panel composed of ST2 and REG3α was found to be able to identify patients at high risk for lethal aGVHD and NRM with an AUC of 0.68, SEN of 40% and SPE of 83% (63). Including also cells to sIL-2R, a 5-parameter biomarker score based on CD4+ T cells, CD8+ T cells, CD19− CD21+ precursor B cells, CD4/CD8 T cell ratio, and sIL-2R was used to predict GVHD onset with AUC of 0.90, SEN of 88.2% and SPE of 66.7% (64). Aiming to identify and evaluate candidate biomarkers potentially predictive of response to treatment with itacitinib plus corticosteroid in aGVHD, plasma monocyte-chemotactic protein (MCP)3, pro-calcitonin/calcitonin (ProCALCA/CALCA), REG3α, ST2, and TNFR1 were found (65).

aGVHD treatment relies on corticosteroid immunosuppression, with initial response guiding further decisions. Analysis of 507 patients from 17 Mount Sinai Acute GVHD International Consortium (MAGIC) centers revealed a validated biomarker algorithm, consisting of ST2 and REG3α measured by ELISA, predicting outcomes in steroid resistant GVHD. MAGIC biomarker probabilities after 1 week of systemic GVHD treatment outperform clinical criteria (AUC 0.82), aiding in the development of improved treatment strategies (66).

### 3.2 Protein based biomarkers for cGVHD

cGVHD usually starts more than 3 months after a transplant and can last for as long as a lifetime (67). Preclinical studies and translational research on human biospecimen have revealed some possible biological pathways in cGvHD. This has opened the way for the exploration of diagnostic, prognostic and predictive biomarkers in both hypothesis-driven and discovery-based testing (68). Biomarkers for cGVHD are presented in **Table 2**.

**Table 2:** Diagnostic and prognostic plasma and serum biomarkers of cGVHD.

Biomarker	Specimen	Method	Clinical relevance	SEN/SPE	AUC	HR	Ref.
Diagnostic biomarker							
CXCL9	Plasma	antibody microarray	**CXCL9** is a significant diagnostic biomarker for cGVHD.	–	0.83	–	(69)
IL-10	Plasma	ELISA	High plasmatic levels of **IL-10** have been associated with the presence of cGVHD.	–	–	–	(70)
Fibronectin	Plasma	ELISA	The level of **plasma fibronectin**, a ligand of CD29, correlated with the number of IL-10 spot-forming cells.	–	–	–	(70)
CD29	Plasma	ELISA	**CD29** expression on monocytes in patients with active cGVHD was elevated.	–	–	–	(70)
CXCL9	Serum	ELISA	**CXCL9** predicts development of severe cGVHD.	–	–	1.33	(71)
Prognostic biomarker							
sBAFF	Plasma	ELISA	**sBAFF** were found to correlate with the severity of cGVHD, but also with the presence of autoimmune disease.	–	–	–	(72)
			**sBAFF** levels at the time of cGVHD diagnosis were also associated with NRM and could be potentially useful for risk stratification.	–	–	5	(73)
CD13	Plasma	ELISA	The presence of the soluble and active form of this protein in the plasma is correlated with cGVHD. Currently, the implication of **CD13** in cGVHD is being studied and the possibility that actinonin, a specific **CD13** inhibitor, can be used as therapeutic agent.	–	–	–	(74)
			High **CD163** concentration was associated with a higher cumulative incidence of *de novo*-onset cGVHD.		0.73		(75)

SEN, sensitivity; SPE, specificity; -, not available; HR, hazard ratio; Ref, reference.

#### 3.2.1 Biomarker panels for cGVHD

Besides the identification of individual biomarkers for cGVHD, efforts are being made to validate panels of biomarkers (**Table 3**), with a much higher accuracy of diagnosis or prognosis.

**Table 3:** MS-based biomarker panels for diagnosis and prediction of cGVHD.

Biomarker panel	Specimen	Clinical relevance	Ref.
sBAFF, anti-dsDNA antibody, sIL-2Rα, and sCD13	Plasma	Useful for diagnosis, early response evaluation, disease management.	(76)
ST2, OPN, MMP3, CXCL9	Plasma	Useful for diagnosis, severity prediction, and NRM.	(77)
cGvHD_MS14 comprising 14 differentially excreted peptides, including fragments from thymosin β-4, eukaryotic translation initiation factor 4γ2, fibrinogen β-chain, or collagens.	Urine	This pattern facilitates the prediction of cGVHD with a SEN and SPE of 84% and 76%, respectively.	(78)
		When integrated with relevant clinical variables in a logistic regression model, the SEN increased to 93%.	
		Notably, cGvHD_MS14 allowed for the prediction of cGVHD up to 55 days before clinical diagnosis and did not recognize acute GvHD. Serving as an independent diagnostic criterion, this pattern showed the potential for early therapeutic intervention	

Ref, reference.

### 3.3 Protein based biomarkers for site-specific involvement in GVHD

**Figure 2:**
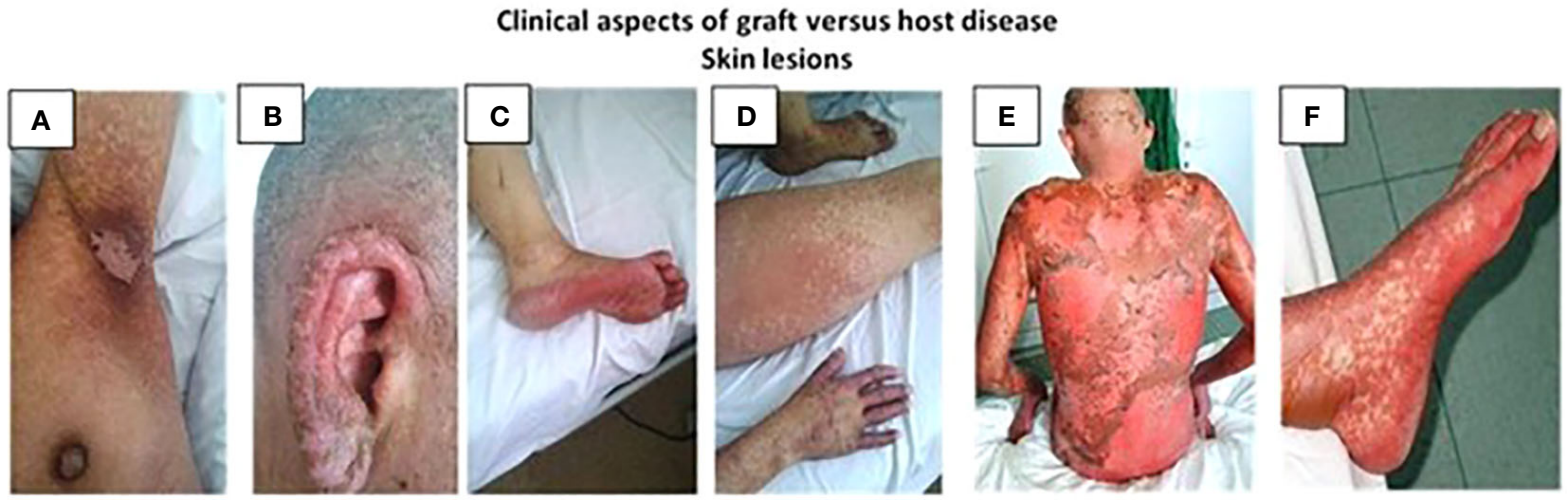
Clinical aspects of GVHD skin lesions **(A)** axillar region; **(B)** ear; **(C)** foot; **(D)** calfs; **(E)** thorax; **(F)** feet.

Site-specific involvement represents a critical facet of GVHD and manifests in distinct anatomical regions, such as the skin, gastrointestinal tract, the eyes, and the lungs, with varying degrees of severity. The skin, often the initial site of presentation, can exhibit rashes, blistering, and itching (79). Clinical aspects of skin lesions in GVHD are illustrated in **Figure 2**.

Ocular symptoms of GVHD can include dry eyes, redness, and sensitivity to light. Oral symptoms of GVHD can manifest as painful mouth sores and difficulty in swallowing (80). Gastrointestinal GVHD may lead to diarrhea, abdominal pain, and even malabsorption. Liver involvement may result in elevated liver enzymes and jaundice. Cough, shortness of breath, and lung inflammation are frequent manifestations of pulmonary site involvement of GVHD (36).

Understanding the site-specific manifestations of GVHD is essential for early diagnosis and tailored treatment strategies, as the severity and response to therapy can differ significantly depending on the affected organ. In this context, site-specific involvement represents a critical aspect of managing GVHD to optimize patient outcomes, one potential option being the protein-based biomarker deciphered below.

#### 3.3.1 Protein biomarkers of GVHD with skin involvement

Tissue biopsies can help identify useful GVHD biomarkers, that can ultimately be sought in biofluids that can be sampled in a less invasive way. For example, skin biopsies can be used for skin GHVD biomarker identification.

Elafin, also known as skin-derived antileukoprotease (SKLP), is an inhibitor of epidermic protease induced by TNF-α and identified at the level of inflamed epidermis in autoimmune diseases such as psoriasis (1, 81). *Mahabal et al.* used skin biopsies and revealed that tissue elafin could be a useful biomarker of aGVHD with skin manifestations (SEN and SPE of 100% and 75%, respectively) (82). Additionally, proteomics studies have highlighted the potential of elafin as a plasmatic biomarker for skin GVHD (83), although the results may be controversial and need further evaluation in larger patient groups (84). *Paczesny et al.* found by quantitative liquid chromatography coupled with MS and further ELISA validation that elafin exhibited increased expression in GVHD skin biopsies. Elevated plasma elafin levels at skin GVHD onset, correlating with the maximum GVHD grade, were linked to a higher mortality risk (HR 1.78). This underscores the diagnostic and prognostic significance of elafin as a biomarker for skin GVHD (85).

#### 3.3.2 Protein biomarkers of GVHD with ocular involvement

Another non-invasive biospecimen, the tears, is in the spotlight of researchers (86, 87). In a recent study focused on cGVHD with ocular symptoms, a team of researchers identified a tear cytokine panel comprising IL-8, IL-10, IFN-γ, CXCL9, CCL17, and CCL19 as promising biomarkers for early diagnosis of ocular GVHD. Moreover, the levels of IL-10, IFN-γ, and CXCL9 were found to be indicative of the severity of ocular GVHD following allo-HSCT (AUCs > 0.6) (88). Furthermore, in a separate investigation, the prognostic role of cytokine levels in tears prior to HSCT was examined in the context of ocular cGVHD. This pilot study included 25 patients who were prospectively monitored, with 19 cytokines measured using a multiplex bead assay. A multistate model, considering four states (HSCT, systemic cGVHD, ocular cGVHD, and death), was constructed to pinpoint cytokines linked to each transition probability. Fractalkine, IL-1Rα, and IL-6 emerged as key contributors with optimal prognostic value (AUC 67% to 80%) for predicting the development of ocular cGVHD after HSCT (89). In advancing understanding of ocular GVHD, a predictive model was developed by multiplex-bead assay at the tear molecule level. Analyzing a cytokine panels correlation with clinical features, the best model, incorporating IL-8/CXCL8, IP-10/CXCL10 tear levels, age, and sex, achieved an AUC of 0.9004, with 86.36% SEN and 95.24% SPE (87). Presence and role of autoantibodies after stem-cell transplantation and their association with cGVHD was also addressed (90) and anticardiolipin antibodies high plasmatic levels have been found to correlate with cGVHD with ocular involvement (91).

#### 3.3.3 Protein biomarkers of GVHD with oral involvement

Because both aGVHD and cGVHD may present with oral alterations such as gingivitis, mucositis, xerostomia, mucosal atrophy, and ulcers, saliva is a target biofluid used in precision medicine approaches. Modification in salivary function is irreversible and reflects systemic GVHD, making saliva sampling and analysis a useful source of diagnostic biomarkers. Salivary proteins with potential biomarker role for oral cGVHD have been studied by MS and confirmed by ELISA or targeted label-free quantification. IL-1Rα and cystatin B distinguished oral cGVHD with a SEN of 85% and SPE of 60%. Particularly, in newly diagnosed patients assessed within 12 months of allo-HSCT, the markers exhibited enhanced discrimination (SEN 92% and SPE 73%) (92). A decline in salivary lactoperoxidase, lactotransferrin, and various proteins within the cysteine proteinase inhibitor family indicated compromised oral antimicrobial host immunity in patients with cGVHD (93). Also, high levels of lactoferrin and lactoperoxidase have been identified in the saliva of patients with GVHD with oral cavity involvement (92).

#### 3.3.4 Protein biomarkers of GVHD with gastrointestinal involvement

GVHD with gastrointestinal involvement has been considered the major cause of morbidity, compared to all the other GVHD manifestations. Several biomarkers have been evaluated for their utility in gastrointestinal GVHD. Recently, the Reg3α has been identified as a potential biomarker for GVHD with intestinal involvement. Reg3α is a protein involved in cellular differentiation and proliferation and in defense against Gram-positive infections of the intestinal tract (1). Quantitative LC-MS analysis identified plasma Reg3α as a valuable predictor for diagnosing gastrointestinal aGVHD. Produced by Paneth cells in the gastrointestinal tract, Reg3α serves as an antimicrobial peptide with bactericidal properties against Gram-positive bacteria and antiapoptotic effects for Paneth cells (94). IL-22, secreted by lymphoid cells in the crypts of the gastrointestinal tract, stimulates Reg3α secretion. In the context of gastrointestinal GVHD, damage to the intestinal mucosa releases Reg3α into the bloodstream, making it a biomarker for gastrointestinal GVHD. Utilizing quantitative LC-MS, the predictive capability of plasma Reg3α for diagnosing gastrointestinal aGVHD was confirmed (AUC 0.80). Furthermore, combining Reg3α with clinical stage and histologic grade enhances risk stratification for patients (95).

Additionally, elevated plasma levels of TIM3, IL-6, and sTNFR1 demonstrated predictive value for the development of peak grade 3-4 GVHD (AUC 0.88). Plasma ST2 and sTNFR1 served as predictors for NRM within 1-year post-transplantation (AUC 0.90). In a landmark analysis, plasma TIM3 predicts subsequent grade 3-4 GVHD (AUC 0.76). Thus plasma levels of TIM3, sTNFR1, ST2, and IL-6 proved to be valuable in predicting more severe GVHD and NRM (96). In liver GVHD without gastrointestinal involvement, a rare occurrence (3% of GVHD patients), REG3α and HGF concentrations were elevated compared to asymptomatic patients but were like liver gastrointestinal GVHD, non-GVHD hyperbilirubinemia, and isolated skin GVHD. KRT18 concentrations were significantly higher in liver GVHD patients than in others, except those with non-GVHD liver complications. However, none of the three biomarkers effectively distinguished liver GVHD from non-GVHD liver complications. Including patients with concomitant gastrointestinal and liver involvement at GVHD onset, REG3α emerged as a stronger diagnostic biomarker for liver gastrointestinal GVHD compared to HGF and Keratin 18 (KRT18). REG3α, HGF, and KRT18 predicted day 28 nonresponse to therapy, while REG3α and HGF are good prognostic markers for 1-year NRM in liver-gastrointestinal GVHD patients (97).

The predictive value of two biomarkers, ST2 and Reg3α, in the case of NRM and GVHD, in allotransplant patients was highlighted. High concentrations of the two biomarkers tested in day 7 after transplant were associated with high GVHD related mortality and more frequent intestinal GVHD, demonstrating their usefulness in the prediction of GVHD before the clinical signs appear (63). Also, another study showed that higher levels of plasma Reg3α were found in patients with gastrointestinal-cGVHD, suggesting the utility of Reg3a as a prognostic biomarker of gastrointestinal-cGVHD (98).

Besides plasma, one more potentially useful source of biomarkers in the context of gastrointestinal GVHD is feces. Intestinal inflammation can be observed by accessing markers of leukocyte activation into the mucosa. A trial aimed to assess the dynamics of fecal biomarkers calprotectin and α1-antitrypsin (α1-AT) in GVHD. Steroid-refractory patients initially exhibited higher biomarker levels, which consistently increased throughout GVHD progression. In cortico-sensitive GvHD, calprotectin and α1-AT showed low and decreasing levels. Second-line treatment in refractory patients did not have predictive biomarker levels, but subsequent responders showed a progressive decrease in calprotectin, while non-responders maintained high levels. α1-AT values had a weaker correlation with treatment response, remaining elevated in both non-responders and responders. Monitoring calprotectin levels proved to be beneficial in managing immunosuppressive treatment for gastrointestinal GVHD (99). While there was a noticeable trend towards elevated serum calprotectin levels in GVHD development and gut involvement, statistical significance was not achieved, unlike fecal calprotectin. Therefore, fecal calprotectin, rather than serum calprotectin, may be considered a potential biomarker for gut GVHD as shown by *Metafuni et al.* (100).

#### 3.3.5 Protein biomarkers of GVHD with pulmonary involvement

Pulmonary cGVHD can present with obstructive and/or restrictive disease. Severity ranges from subclinical pulmonary impairment to respiratory insufficiency with bronchiolitis obliterans, a feature of pulmonary cGVHD. Early diagnosis may improve clinical outcome, and regular post-transplant follow-ups are recommended (101).

In recent proteomics studies, regulatory proteins of the extracellular matrix have been identified as candidate biomarkers for the diagnostic and prognosis of bronchiolitis obliterans syndrome (BOS) and GVHD with pulmonary involvement. Plasmatic levels of matrix metalloproteinase 3 (MMP3) have been associated with the presence of BOS and could be correlated with disease severity (102). In another study, the diagnostic value of the lead candidate, MMP3, was evaluated by ELISA in plasma and was found to differ significantly between patients with and without BOS (AUC 0.77) (102). Moreover, in another study, the specific BOS biomarker potential of osteopontin (OPN) was explored in patients with cGVHD. Using immunohistochemistry, *Williams et al.* showed that elevated OPN plasma levels observed in patients with BOS, potentially originating from alveolar macrophages, correlated with disease severity. These findings, supported by lung immunohistochemistry, could be valuable for diagnosing and prognosing BOS after HSCT (103).

## 4 Conclusions

In summary, the exploration of protein-based biomarkers for GVHD post-allo-HSCT is crucial for improving diagnostic accuracy and treatment outcomes. Proteomics, with its ability to increase the potential of minimally invasive biopsies, serves as a powerful strategy for biomarker discovery by analyzing intricate protein profiles. Proteins, ranging from cytokines to emerging candidates, play a pivotal role as valuable biomarker sources. The strategies outlined, such as focusing on cytokine-based biomarkers, emerging protein-based markers, and biomarker panels for acute and chronic GVHD, as well as for site specific involvement of GVHD, offer a roadmap for future research, promising improved diagnostic precision and personalized treatment approaches. Nevertheless, the existing literature on GVHD protein-based biomarkers is characterized by substantial heterogeneity in reports, a lack of standardized study protocols, and inconsistent inclusion of patients. This heterogeneity and variability in methodology represent significant challenges that contribute to the observed disparity between the multitude of promising biomarkers identified in individual studies and the limited number of biomarkers poised for clinical translation. Consequently, the immediate focus may not solely be on the discovery of novel molecules and pathways, but rather on the critical task of validating or exposing the efficacy of existing methods. Addressing these challenges is imperative for bridging the gap between bench side research and clinical application in the near term.

However, the integration of proteomics into GVHD research not only enhances our understanding of the disease but also positions us at the forefront of transformative advancements in clinical practice. Collaborative efforts and technological advancements in proteomic analysis are essential for realizing the full potential of protein-based biomarkers, paving the way for a future where GVHD diagnosis and treatment are increasingly personalized and effective.

## Author contributions

MI: Writing – original draft, Writing – review & editing. CP: Writing – original draft, Writing – review & editing. AU: Writing – original draft, Writing – review & editing. CM: Writing – original draft, Writing – review & editing. DC: Writing – original draft, Writing – review & editing. MZ: Writing – original draft, Writing – review & editing. AT: Writing – original draft, Writing – review & editing. JB: Writing – original draft, Writing – review & editing. VG: Writing – original draft, Writing – review & editing. MC: Writing – original draft, Writing – review & editing. CI: Writing – original draft, Writing – review & editing, Conceptualization, Formal analysis, Funding acquisition, Resources, Supervision. CT: Writing – original draft, Writing – review & editing, Conceptualization, Formal analysis, Resources, Supervision. DT: Writing – original draft, Writing – review & editing, Conceptualization, Formal analysis, Supervision.
